# Dr. H. W. Carpenter

**Published:** 1907-06

**Authors:** 


Dr. H. W. Carpenter, of Oneida, N. Y., died at his home in that city May 19, 1907. He graduated at the Medical Department, New York University, in 1858. He had served as member of assembly and as coroner of Madison County.

